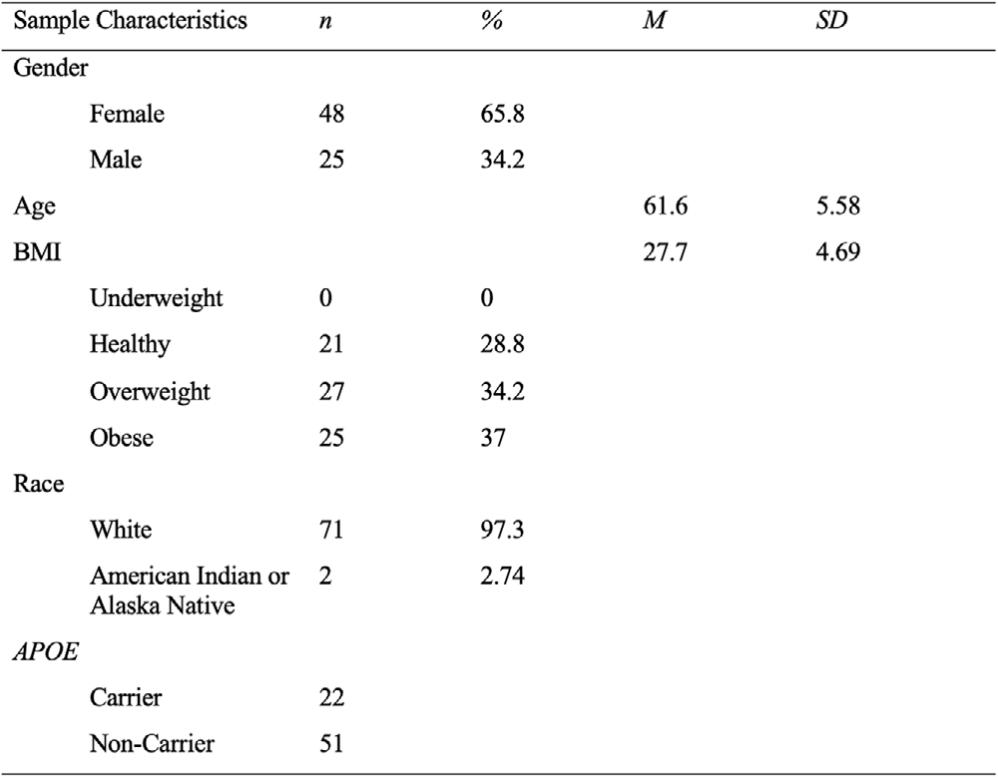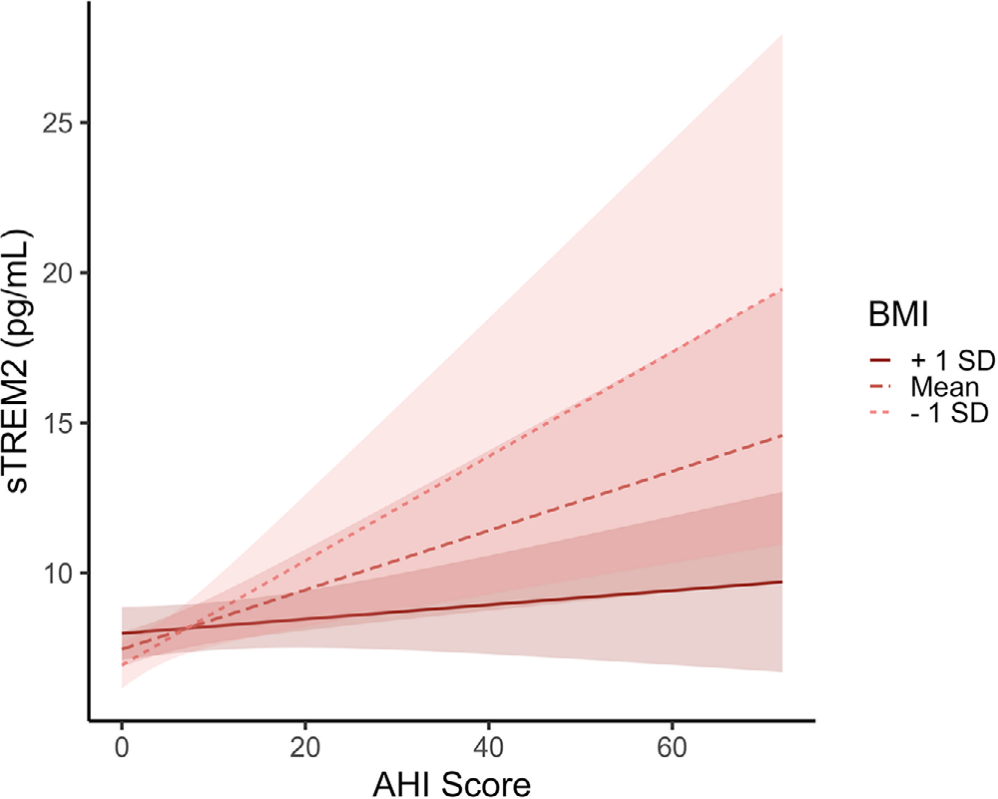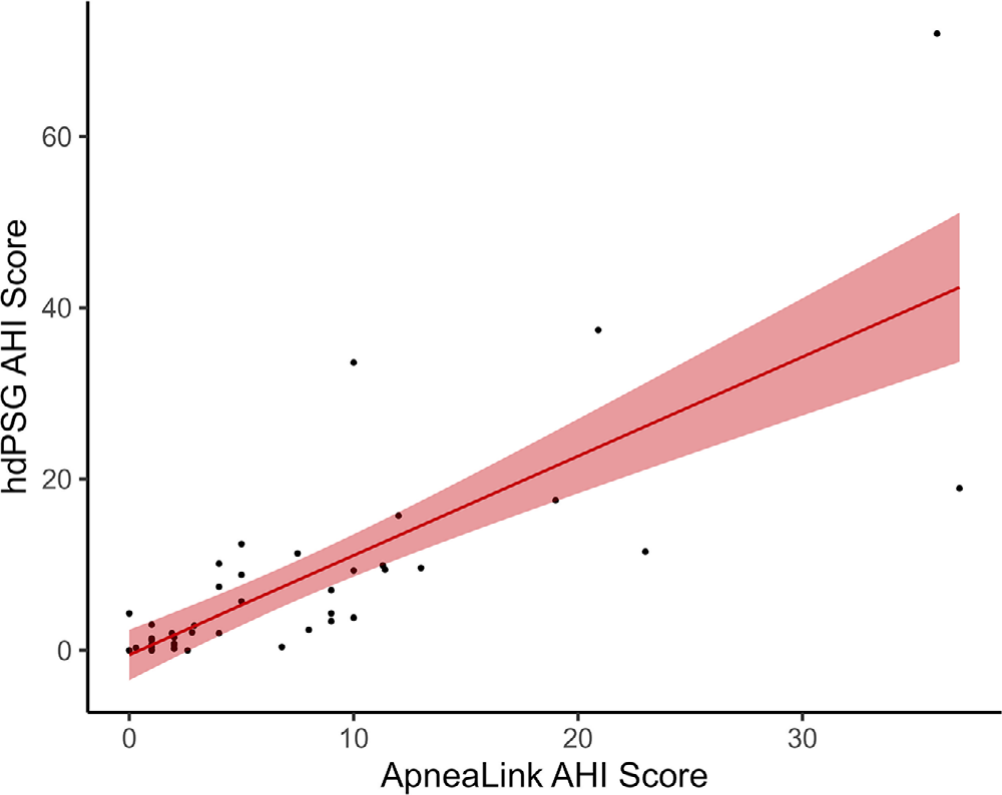# Body mass index moderates the relationship between the apnea‐hypopnea index and CSF biomarker of activated microglia

**DOI:** 10.1002/alz.095742

**Published:** 2025-01-09

**Authors:** Julia Bink, Yue Ma, Bryce A Mander, David T Plante, Margherita Carboni, Norbert Wild, Nathaniel A. Chin, Ozioma C Okonkwo, Cynthia M. Carlsson, Carey E. Gleason, Sanjay Asthana, Sterling C. Johnson, Kaj Blennow, Henrik Zetterberg, Ruth M Benca, Barbara B. Bendlin

**Affiliations:** ^1^ University of Wisconsin ‐ Madison, Madison, WI USA; ^2^ Wisconsin Alzheimer’s Disease Research Center, University of Wisconsin School of Medicine and Public Health, Madison, WI USA; ^3^ University of California Irvine, Orange, CA USA; ^4^ University of Wisconsin‐Madison, Madison, WI USA; ^5^ Roche Diagnostics International Ltd, Rotkreuz, Zug Switzerland; ^6^ Roche Diagnostics International Ltd, Rotkreuz Switzerland; ^7^ Alzheimer’s Disease Research Center, University of Wisconsin‐Madison, Madison, WI USA; ^8^ Wisconsin Alzheimer’s Disease Research Center, School of Medicine and Public Health, University of Wisconsin‐Madison, Madison, WI USA; ^9^ Alzheimer’s Disease Research Center, University of Wisconsin‐Madison School of Medicine and Public Health, Madison, WI USA; ^10^ University of Gotenborg, Gothenburg Sweden; ^11^ Institute of Neuroscience and Physiology, University of Gothenburg, Gothenburg, Mölndal Sweden; ^12^ Wake Forest School of Medicine, Wake Forest University, Winston‐Salem, NC USA; ^13^ Wisconsin Alzheimer’s Disease Research Center, University of Wisconsin‐Madison, School of Medicine and Public Health, Madison, WI USA

## Abstract

**Background:**

Prior studies suggest that obstructive sleep apnea (OSA) may be associated with Alzheimer’s disease (AD) pathology, including Aβ42/Aβ40 and p‐Tau181. However, less is known about relationships between OSA and non‐AD pathology, including neurofilament light chain (NfL), glial fibrillary acidic protein (GFAP), S100, chitinase 3‐like 1 (YKL40), and soluble triggering receptor expressed on myeloid cells 2 (sTREM2), as well as the effect of potential moderating factors. The present study investigated the relationship between the apnea‐hypopnea index (AHI) and cerebrospinal fluid (CSF) biomarkers of AD and related pathology.

**Method:**

In this cross‐sectional study, 73 cognitively unimpaired adults with no prior OSA diagnosis underwent an overnight PSG and/or testing with an at home ApneaLink. Participants completed a lumbar puncture to determine Aβ42/Aβ40, pTau181, NfL, GFAP, s100, YKL‐40, and sTREM2 as part of the Roche NeuroToolKit research platform (Roche International). Relationships between AHI and CSF biomarkers were tested using multiple linear regression adjusting for age, sex, *APOE* genotype, and BMI. Given prior studies suggesting potential interaction effects, we also examined interactions between AHI and *APOE* and interactions between AHI and BMI.

**Result:**

AHI determined using PSG and at home Apnealink were highly correlated (r = 0.78, p < 0.01). There were no significant main effects of AHI on any of the CSF biomarkers, controlling for covariates (p > 0.05). Further, there was no significant interaction between *APOE* and AHI (p > 0.05). We found a significant interaction between BMI and AHI, such that participants with lower BMI showed higher sTREM2 with greater AHI, (p = 0.02; Figure 1). However, this positive relationship diminished with higher BMI.

**Conclusion:**

OSA and obesity are intrinsically linked processes, however prior studies in sleep have also shown that these factors have interactive effects on several outcomes, including measures of inflammation and cognitive function. Here, we found that individuals who had lower BMI showed a potentially deleterious effect of AHI on measures of microglial activation. Additional studies are needed to determine whether these results are due to possible protective effects of higher BMI, or differences in sleep quality among individuals with high and low BMI.